# Superior adjuvanticity of the genetically fused D1 domain of *Neisseria meningitides* Ag473 lipoprotein among three Toll-like receptor ligands

**DOI:** 10.1042/BSR20193675

**Published:** 2020-04-07

**Authors:** Huipeng Lu, Xiaokai Zhang, Yuyang Wang, Yang Zong, Yajie Wang, Xinyu Zhang, Xiaoli Xia, Huaichang Sun

**Affiliations:** 1 College of Veterinary Medicine, Jiangsu Co-Innovation Center for Prevention and Control of Important Animal Diseases and Zoonoses, Yangzhou University, Yangzhou 225009, China; 2 The Testing Center, Yangzhou University, Yangzhou 225009, China; 3 Jiangsu Agri-animal Husbandry Vocational College, Taizhou 225300, China

**Keywords:** Adjuvanticity comparison, Different delivery ways, Toll-like receptor ligands

## Abstract

Toll-like receptor (TLR) ligands have emerged as the attractive adjuvant for subunit vaccines. However, selection of TLR ligands needs to be rationally chosen on the basis of antigen and adjuvant properties. In the present study, we expressed the Ag473 lipoprotein from *Neisseria meningitides*, flagellin FlaB from *Vibrio vulnificus* and heat shock protein 70 from *Mycobacterium tuberculosis* (mHsp70) in *Escherichia coli* as single proteins and fusion proteins with VP2 protein of infectious bursal disease virus (IBDV). Both cellular and humoral adjuvanticities of the three TLR ligands were compared by immunization of mice in two different ways. Among the three co-administered TLR ligands, recombinant Ag473 lipoprotein exhibited the highest cellular and humoral adjuvanticities, including promotion of IL-4, IL-12, IFN-γ and IBDV VP2-specific antibody production. Among the three genetically fused TLR ligands, fusion with Ag473 D1 domain exhibited the highest cellular and humoral adjuvanticities. Overall, the adjuvanticities of genetically fused TRL ligands were significantly higher than that of co-administered TLR ligands. Fusion with Ag473 D1 domain exhibited superior adjuvanticity among the three TLR ligands delivered in two different ways.

## Introduction

Recombinant protein- or synthetic peptide-based subunit vaccines have a better safety profile than traditional inactivated and live attenuated vaccines. However, these well-defined vaccine candidates are usually poorly immunogenic and thus require appropriate adjuvant to be efficacious [1]. Although alum salts and oil-in-water emulsions are still commonly used in licensed vaccines due to their good safety record, these traditional adjuvants cannot stimulate appropriate cellular immune response that is important for antiviral infections [2]. Therefore, this is an urgent need to develop novel adjuvants that can shape vaccine-induced immune responses.

Toll-like receptors (TLRs) play critical roles in both innate and adaptive immune responses. Among 13 TLRs known in mammals, TLR1, 2, 4, 5 and 6 are expressed on the cell surface to recognize bacterial membrane components, while TLR3, 7, 8 and 9 present in the intracellular compartments are involved in detecting viral nucleic acids [3]. By promoting cellular activation, cytokine production and/or the maturation of professional antigen-presenting cells (APC), TLR ligands can stimulate both innate and adaptive immune responses [4]. Therefore, targeting subunit vaccines to TLR agonists has become a hot field of vaccine research.

Bacterial flagellins and lipoproteins are the two most-studied TLR5 and TLR2 agonists, respectively. The D1 domain of Ag473 lipoprotein of *Neisseria meningitides* contains the characteristic bacterial lipoprotein signal peptide and lipobox with an invariant cysteine for lipid modification [5]. Fusion with the D1 domain of Ag473 lipoprotein can convert dengue virus E3 envelope protein into a highly immunogenic lipoprotein. A single-dose immunization of mice with the lipodated antigen without extra adjuvant formulation is sufficient to elicit neutralizing antibodies against all four serotypes of dengue viruses [6]. The same strategy has been used to produce novel subunit vaccines against *Clostridium difficile*-associated diseases and HPV-based immunotherapeutic vaccines [7]. The FlaB flagellin of *Vibrio vulnificus* has highly potent mucosal adjuvant activity. By intranasal immunization of mice with FlaB flagellin and tetanus toxoid, the antigen-specific IgA and IgG responses are significantly enhanced, leading to the complete protection against a lethal dose of tetanus toxin challenge [8]. Furthermore, intranasal immunization of mice with FlaB flagellin and the P domain of VP1 protein from *Norovirus* can induce a potent antibody and cellular immune responses in both systemic and mucosal compartments [9]. Fusion of the ectodomain of matrix protein M2 from influenza virus to the C-terminus of heat shock protein 70 from *Mycobacterium tuberculosis* (mHsp70) can result in more potent and protective responses in the absence of adjuvants. A prime-boost administration of mice with the mHsp70-fused antigen provides full protection against lethal dose of mouse-adapted H1N1, H3N2 or H9N2 influenza A isolates [10].

As the adjuvants for subunit vaccines, TLR ligands can be either co-administered or genetically fused with vaccine antigens. The genetic fusion strategy can ensure the co-localization of antigen and adjuvant to the same APC, thereby enhancing the antigen presentation and processing efficiency [1]. However, the selection of these approaches needs to be rationally chosen on the basis of antigen and adjuvant properties [11]. In the present study, we compared the adjuvanticities of three different TLR ligands delivered in two different ways using the recombinant VP2 protein of infectious bursal disease virus (IBDV) as the model antigen in a mouse model.

## Materials and methods

### Bacterial strains

*Escherichia coli* strains DH5α and BL21 (DE3) (Addgene, U.S.A.) were used as the host strains for routine gene cloning and expression experiment, respectively.

### Animals

All animal experiments were performed in accordance with the guidelines and animal use protocols approved by the Institutional Animal Care and Use Committee, Yangzhou University. Female BALB/c mice (6 or 8 weeks old) were purchased from the Comparative Medicine Center and housed in the animal biosafety level 2 facility at the College of Veterinary Medicine, Yangzhou University.

### Vector construction

The coding sequences for the immunodominant segment of VP2 protein from IBDV [12], C-terminal segment of mHsp70 (GenBank accession: ACE79189.1), major flagellin FlaB from *Vibrio vulnificus* (GenBank accession: WP_011078329.1) and Ag473 lipoprotein from *Neisseria meningitides* (GenBank accession: WP_002220617.1) were adapted to *E. coli* codon usage using JAVA Codon Adaption Tool [13]. The synthetic sequences were cloned individually into pET-30a (+) vector (Novagen, U.S.A.) with *Nde*I and *Xho*I digestion. Then, the coding sequences for the VP2 segment, Ag473 D1 domain, FlaB and mHsp70 were amplified by PCR using primer pairs listed in Supplementary Table S1. The *VP2* amplicon was cloned into pET-30a (+) vector with *Bam*HI/*Xho*I digestion. The *Ag473 D1* or *FlaB* amplicon was cloned into the *VP2*-containing vector with *Nde*I/*Bam*HI digestion. The *mHsp70* amplicon, with a (GGGS)_3_ linker at the 5-end, was cloned into the *VP2*-containing vector with *Nco*I/*Xho*I digestion. The recombinant vectors confirmed by sequence analysis (Sangon Biotech, China).

### Protein expression and purification

The above recombinant vectors were transformed individually into *E. coli* strain BL21 (DE3). After overnight growth on agar plates containing kanamycin (50 μg/ml), single colonies were grown overnight in kanamycin-containing Luria-Bertani (LB) medium. Each of the overnight cultures was inoculated into 2 × YT medium (10 g/l yeast extract, 16 g/l tryptone, 5 g/l NaCl, pH 7.4), and grown to OD_600_ of 0.8 at 37°C. The expression of recombinant proteins was induced with 0.2 mM isopropyl β-D-thiogalactoside (IPTG) for 6 h at 37°C. The expressed proteins were purified using Ni-NTA agarose resin (CWBIO, China) by following the manufacturer’s instruction. The purified recombinant proteins were analyzed by 12% SDS-PAGE.

### Western blotting

Since all of the recombinant proteins have His-tags at their C-termini, the purified proteins were identified by Western blotting using His-tag specific mAb (1:1000) (Sangon Biotech, China) as the first antibody and DyLight^800^-labeled goat anti-mouse IgG (1: 10,000) (SeraCare Life Sciences, U.S.A.) as the second antibody. The hybridization signals were scanned using Odyssey Infrared Imaging System (LI-COR Biosciences, U.S.A.).

### MALDI TOF/TOF mass spectrometry

Lipid modification of Ag473 D1-VP2 fusion protein was analyzed using AB5800 MALDI-TOF/TOF mass spectrometry (Applied Biosystem, U.S.A.) as previously described [6,14]. First, the amino acid sequence of D1-VP2 fusion protein was analyzed using peptide characterization software PeptideMass (https://web.expasy.org/peptide_mass) to obtain the predicted tryptic peptides. Then, the purified fusion protein was dialyzed against 5 mM ammonium bicarbonate (pH 8.5), and mixed with trypsin at a ratio of 50:1 (w/w). After overnight digestion at 37°C, the digestion was stopped with α-cyano-4-hydroxycinnamic acid. The N-terminal tryptic segment was analyzed using a 5800 MALDI TOF/TOF analyzer, and the mass spectra (*m/z* 800–4000) were acquired in positive ion reflector mode. In the mass spectrometry (MS) spectrum, the peaks at *m/z* 1452.0, 1466.0 and 1480.0 were selected for subsequent MS/MS sequencing analysis in 2 kV modes.

### Animal immunization

Fifty one BALB/c mice were assigned into eight experimental groups (*n* = 6) and one control group (*n* = 3). Each animal in the experimental groups was immunized intramuscularly with an equal mole of VP2 (20 μg), D1-VP2 (15 μg), FlaB-VP2 (86 μg), VP2-mHsp70 (68 μg), VP2 + Ag473 (10 μg), VP2 + FlaB (10 μg) or VP2 + mHsp70 (10 μg). The control animals were injected with 100 μl of PBS. All experimental animals were boosted with the same antigen formulation at day 14 post immunization (dpi). The serum samples were collected from mice at 7, 14, 21 and 28 dpi for antibody detection, and splenocytes were isolated from immunized mice humanely killed by cervical dislocation at 28 dpi for cytokine detection.

### ELISA

All serum samples from immunized mice were assayed for IBDV VP2-specific antibody titers by indirect ELISA. Briefly, ELISA plates were coated overnight with purified VP2 protein (10 μg/ml) at 4°C. After 1-h blocking with 5% defatted milk powder in PBST (0.01% Tween 20 in PBS) at 37°C, serially diluted serum samples were added and incubated for 1.5 h at 37°C. After washing with PBST, HRP-labeled goat-anti mouse IgG, IgG1 or IgG2b (1: 10,000) (Abcam, U.S.A.) was added and incubation was continued for 1.5 h at 37°C. After 15-min development with 3,3′,5,5′-tetramethylbenzidine (TMB) in dark, the reaction was stopped with 2 M H_2_SO_4_ and OD_450_ values were measured on Super Microplate Reader (Gene Company Ltd, U.S.A.).

### Cytokine assay

All experimental animals were killed by cervical dislocation at 28 dpi for splenocyte isolation. After removing red blood cells with ACK Lysis Buffer (Beyotime Biotechnology, China), splenocytes (5 × 10^6^/ml) were seeded in triplicates on 24-well plates, and cultured overnight in RPMI 1640 medium (HyClone, U.S.A.) supplemented with 10% fetal bovine serum (FBS). After 72-h stimulation with VP2 recall antigen (10 μg/ml), the culture media were analyzed for IL-4, IL-12, TNF-α and IFN-γ production (pg/ml) using Cytokine Detection ELISA Kits (Boster Biological Technology, China) by following the manufacturer’s instruction.

### Statistical analysis

The cytokine and antibody data were analyzed using SPSS 22 software (IBM, U.S.A.), and the mean values and standard deviations (SD) were calculated for each experimental group. The difference between control and experimental groups was compared using one-way analysis of variance (ANOVA) followed by Dunn’s multiple-comparison tests or two-tailed unpaired *t* tests. The *P* values of < 0.05 (*) and < 0.01 (**) were considered to be significant.

## Results and discussion

### Expression and identification of recombinant proteins

To compare the adjuvanticities of three TLR ligands delivered in two different ways, the synthetic sequences for the VP2 protein of IBDV, C-terminal segment of mHsp70, Ag473 lipoprotein or its D1 domain of *N. meningitides*, and flagellin FlaB of *V. vulnificus* were cloned individually or as VP2 fusion genes into pET-30 vector. The recombinant vectors were called pET-VP2, pET-Ag473, pET-FlaB, pET-mHsp70 or pD1-VP2, pFlaB-VP2 and pVP2-mHsp70, respectively. Restriction digestion and sequencing analysis confirmed that all of the seven recombinant vectors were correctly constructed. These recombinant vectors were transformed individually into *E. coli* strain BL21 (DE3), and the expression of recombinant proteins was induced with IPTG. SDS-PAGE analysis showed that an expected of 14- or 42-kDa protein was expressed in pET-VP2 or pET-FlaB recombinant *E. coli*, which was present mainly in the insoluble fraction of centrifuged cell lysate (Supplementary Figure S1A,B). An expected 30- or 32-kDa protein was expressed in pET-mHsp70 or pET-Ag473 recombinant *E. coli*, which was present mainly in the soluble fraction of centrifuged cell lysate (Supplementary Figure S1C,D). An expected 19- or 54-kDa protein was expressed in pD1-VP2 or pFlaB-VP2 recombinant *E. coli*, which was present mainly in the insoluble fraction of centrifuged cell lysate (Supplementary Figure S1E,F). Finally, an expected 45-kDa protein was expressed in pVP2-mHsp70 recombinant *E. coli*, which was present mainly in the soluble fraction of centrifuged cell lysate (Supplementary Figure S1G). SDS-PAGE analysis showed that all of the seven recombinant proteins were purified to almost single bands by using nickel affinity chromatography (Supplementary Figure S1). Western blotting analysis showed that all of the seven recombinant proteins were recognized by His-tag specific mAb (Supplementary Figure S2). Both recombinant Ag473 and its D1 fusion proteins have been expressed in *E. coli* strain C43 (DE3), but not in BL21 (DE3) due to the lower transformation efficiency and higher toxicity [6]. In the present study, however, both recombinant Ag473 and D1-VP2 fusion proteins were expressed efficiently in BL21 (DE3), which may be contributed to adaption of the coding sequence for *E. coli* codon usage.

### Characterization of lipid modification of D1-VP2 fusion protein

To characterize the lipid modification of D1-VP2 fusion protein expressed in BL21 (DE3), the amino acid sequence was analyzed by PeptideMass analysis and 11 tryptic peptides were identified (Supplementary Table S2). After cleavage off Ag473 signal peptide, the N-terminal peptide was expected to be 6-aa long (Supplementary Figure S3A). After digestion with trypsin, the N-terminal peptide was analyzed by MALDI-TOF spectrometry. Three peaks with m/z values of 1452.0, 1466.0 and 1480.0 were revealed (Supplementary Figure S3B). The mass difference between the three peaks were 14 atomic mass units and the peak patterns were almost identical to that of recombinant Ag473 expressed in C43 (DE3) [6]. MALDI-TOF/TOF spectrometry analysis showed that the N-terminal tryptic segment of D1-VP2 fusion protein was also composed of five peaks with *m/z* values of 562.3, 475.2, 347.2, 218.1 and 147.1, respectively, which corresponded to the lipid-modified C-SQEAK sequence of Ag473 lipoprotein (Supplementary Figure S3C). Therefore, BL21-expressed D1-VP2 fusion protein exhibited the characteristics of a lipoprotein with post-translational lipid modifications almost identical to that of Ag473 lipoprotein expressed in *E. coli* strain C43 [6].

### Comparison of cellular adjuvanticities of three TLR ligands

**Figure 1:**
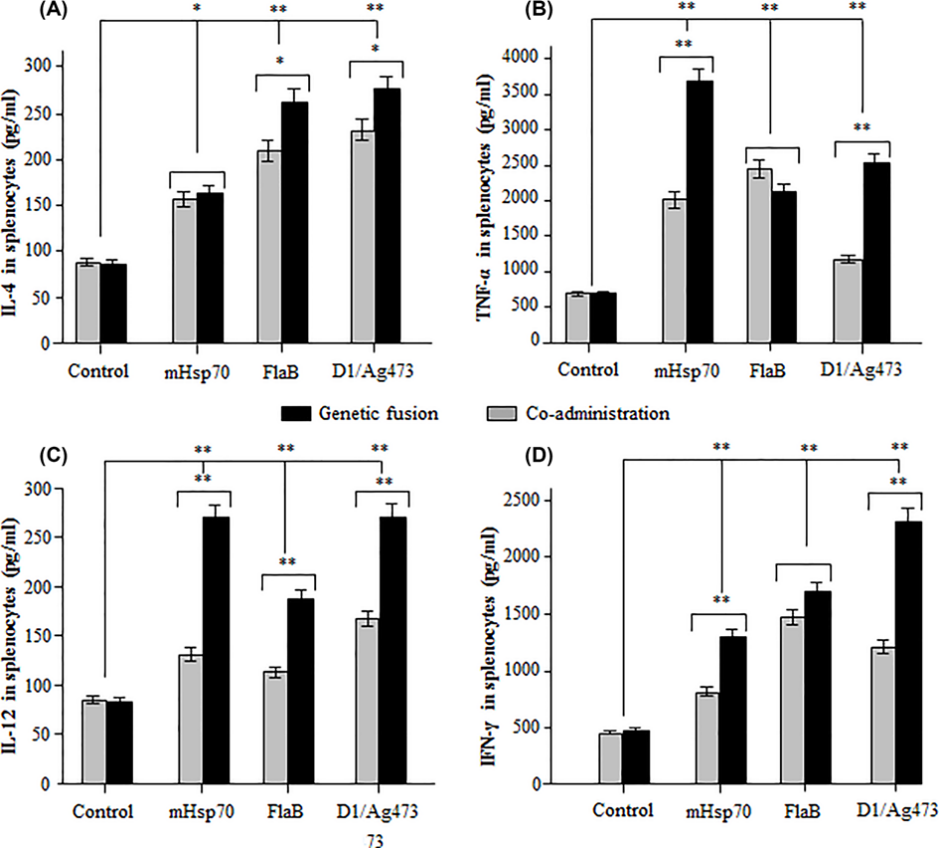
Comparison of the cellular adjuvanticities of three TLR ligands delivered via two different ways Mice were immunized two times with the same dose of indicated antigen/adjuvant combinations, and their splenocytes were isolated at 28 dpi. After 72-h stimulation with VP2 recall antigen, concentrations of IL-4 (**A**), TNF-α (**B**), IL-12 (**C**) and IFN-γ (**D**) in the cell cultures were analyzed using Cytokine Detection ELISA Kits. * or ** indicates *P*<0.05 or *P*<0.01 as compared with the VP2 control or between geneticaly fused and co-administered TLR ligands.

Mice were immunized two times with the seven different antigen formulations. At 28 dpi, the immunized mice were killed for splenocyte isolation. After 72-h stimulation with the VP2 recall antigen, the culture media were harvested for cytokine detection. Compared with the VP2 immunization control, a significantly higher concentration of IL-4 was detected in the splenocytes from mouse immunized with VP2 + Ag473 (*P* < 0.01), VP2 + FlaB (*P* < 0.01), VP2 + mHsp70 (*P* < 0.05), D1-VP2 (*P* < 0.01), FlaB-VP2 (*P* < 0.01) or VP2-mHsp70 (*P* < 0.05). Among them, IL-4 concentrations of D1-VP2 and FlaB-VP2 immunization groups, but not VP2-mHsp70 immunization group, were significantly (*P* < 0.05) than that of VP2 + Ag473 and VP2 + FlaB immuization groups (Figure 1A). Compared with the VP2 immunization control, significantly (*P* < 0.01) higher concentrations of TNF-α, IL-12 and FN-γ were detected in the mouse splenocytes from all of the six adjuvant groups. Among them, IL-12 concentrations of D1-VP2, VP2-mHsp70 and FlaB-VP2 immunization groups were significantly (*P* < 0.01) higher than that of VP2 + Ag473, VP2 + mHsp70 and VP2 + FlaB immunization groups (Figure 1C). Concentrations of TNF-α and FN-γ of D1-VP2 and VP2-mHsp70 immunization groups, but not FlaB-VP2 immunization group, were significantly higher (*P* < 0.01) than that of VP2 + Ag473 and VP2 + mHsp70 (Figure 1B,D).

Generally, signaling via TLR3, TLR4, TLR7, TLR8 and TLR9 promotes Th1-type immune responses, while signaling via TLR2 (along with TLR1 and/or TLR6) and TLR5 favors Th2-type immune responses [15,16]. Th1-type response is defined by pro-inflammatory cytokines IL-12, IFN-γ and TNF-α, while Th2-type response is defined by IL-4, IL-5, IL-6, IL-10 and IL-13 [17]. In the present study, significantly higher levels of Th1 (IL-12, IFN-γ and TNF-α) and Th2 (IL-4) cytokines were detected in mouse splenocytes after immunization with all of the three TLR ligands, genetically fused TLR ligands in particular. The mixed Th1/Th2 response stimulated by Ag473 lipoprotein or its D1 domain can be explained by the interaction with TLR2 along with TLR1 and/or TLR6 [18]. The mixed Th1/Th2 response stimulated by FlaB can be contributed to its ability to activate TLR5-expressing cells through MyD88-independent pathway that involves the formation of TLR5/TLR4 heterodimer [19]. The mixed Th1/Th2 response stimulated by mHsp70 is consistent with its interaction with TLR2 and TLR4 to promote Th1 and Th2 cytokine production [20]. Overall, the cellular adjuvanticities of three genetically fused TLR ligands were much stronger than that of three co-administered TLR ligands. Ag473 D1 domain exhibited the strongest cellular adjuvanticity among the three TRL ligands delivered in two different ways.

### Comparison of humoral adjuvanticities of three TLR ligands

**Figure 2:**
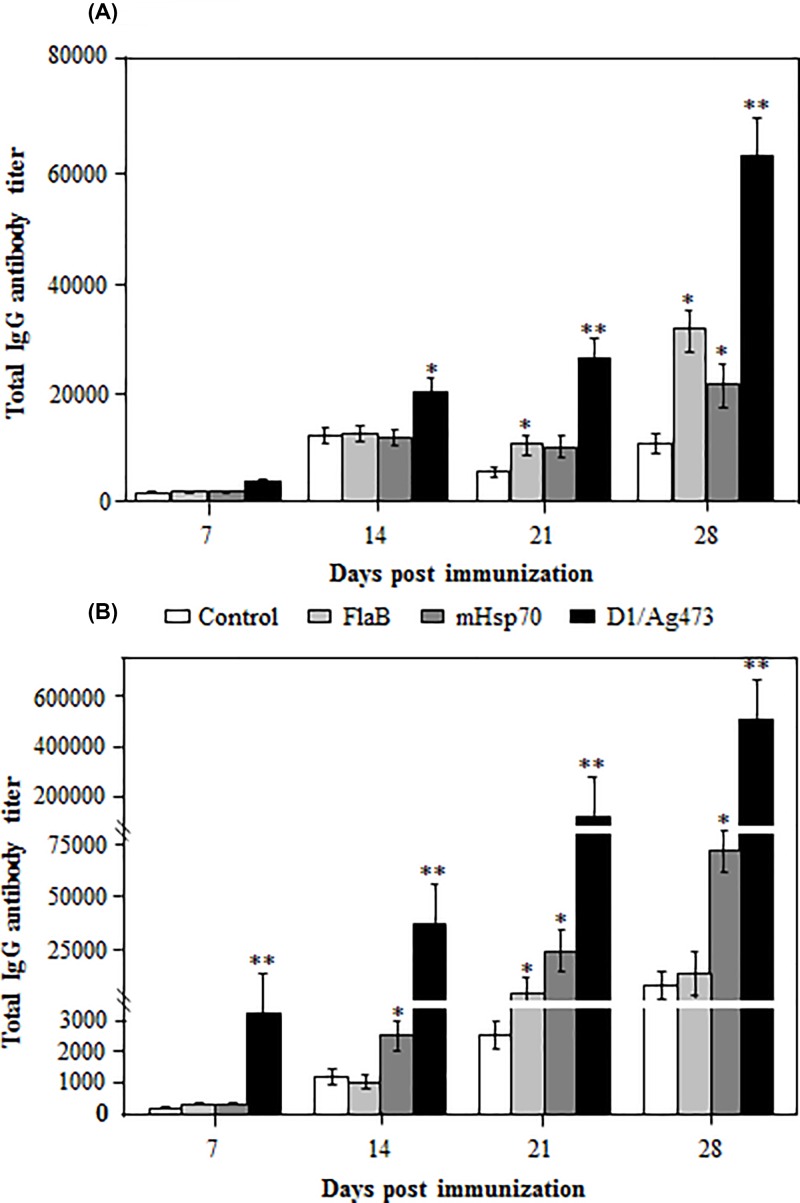
Comparison of the humoral adjuvanticities of three TLR ligands delivered via two different ways Mice were immunized two times with the same dose of indicated antigen/adjuvant combinations, and their serum samples were assayed for the antigen-specific IgG antibody at the indicated times by ELISA using VP2 as the antigen. * or ** indicates *P*<0.05 or *P*<0.01 as compared with the VP2 control or between co-administered (**A**) and geneticaly fused (**B**) TLR ligands.

To compare the humoral adjuvanticities of three co-administerd TLR ligand, mice were immunized with the recombinant VP2 protein without or with three TLR ligands, and the serum samples were collected for antibody detection at different dpi. At 7 dpi, the antigen-specific IgG antibody titer was slightly higher in VP2 + Ag473 immune serum, but not in VP2 + FlaB or VP2 + mHsp70 immune serum as compared with that in VP2 immune serum (Figure 2A). At 14 dpi, a significantly (*P* < 0.05) higher titer of the antigen specific IgG antibody was detected in VP2 + Ag473 immune serum, but not in VP2 + FlaB or VP2 + mHsp70 immune serum (Figure 2A). At 21 dpi, a significantly high level of the antigen specific IgG was detected in VP2 + Ag473 (*P* < 0.01) or VP2 + FlaB immune serum (*P* < 0.05), but not in VP2 + mHsp70 immune serum (Figure 2A). By 28 dpi, the antigen specific IgG titer reached to the highest level in VP2 + Ag473 (*P* < 0.01), VP2 + FlaB (*P* < 0.05) and VP2 + mHsp70 (*P* < 0.05) immune serum (Figure 2A). Among the three co-administered TLR ligands, Ag473 exhibited the highest humoral adjuvanticity, followed by FlaB and mHsp70.

To compare the humoral adjuvanticities of three genetically fused TLR ligand, mice were immunized with the recombinant VP2, D1-VP2, FlaB-VP2 or VP2-mHsp70, and the serum samples were collected for antibody detection at different dpi. As early as 7 dpi, the antigen specific IgG antibody titer was significantly (*P* < 0.01) higher in D1-VP2 immune serum, but not in FlaB-VP2 or VP2-mHsp70 immune serum as compared with that in VP2 immune serum (Figure 2B). At 14 dpi, the antigen specific IgG antibody response increased to a significantly higher level in D1-VP2 (*P* < 0.01) or VP2-mHsp70 (*P* < 0.05) immune serum, but not in FlaB-VP2 immune serum (Figure 2B). At 21 dpi, the antigen specific IgG antibody response increased further in D1-VP2 (*P* < 0.01), FlaB-VP2 (*P* < 0.05) or VP2-mHsp70 (*P* < 0.05) immune serum (Figure 2B). By 28 dpi, the antigen specific IgG antibody response reached to the highest levels in D1-VP2 or VP2-mHsp70 immune serum, but not in FlaB-VP2 immune serum (Figure 2B). Among the three genetically fused TLR ligands, fusion with Ag473 D1 domain exhibited the highest humoral adjuvanticity, followed by fusion with mHsp70 and FlaB.

Generally, the humoral adjuvanticities of three genetically fused TLR ligands were much stronger than that of co-administered TLR ligands. Among the six antigen/adjuvant combinations, fusion with Ag473 D1 domain showed superior adjuvanticity to promote the VP2-specific antibody response, confirming that the lipobox within the D1 domain is responsible for the adjuvanticity of Ag473 lipoprotrein [18]. The genetically fused mHsp70 exhibited significantly stronger adjuvanticity than co-administrated mHsp70, confirming its requirement for physical fusion with the antigen to function as a vaccine adjuvant [10]. Interestingly, both co-administered and genetically fused FlaB showed very low humoral adjuvanticity, which may be explained by its mucosal adjuvanting nature and the need to be administered via mucosal route to function as a vaccine adjuvant [8].

### Further characterization of humoral adjuvanticity of Ag473 D1domain

**Figure 3:**
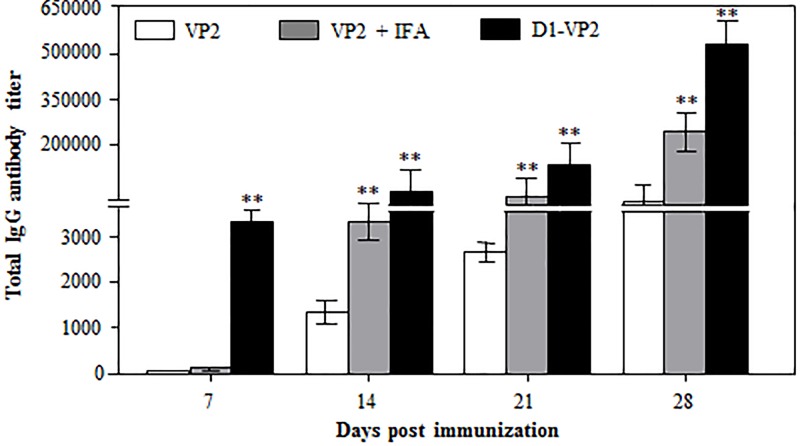
Comparison of humoral adjuvanticities between genetically fused Ag473 D1 domain and IFA Mice were immunized two times with the same mole of indicated antigen formulations, and their serum samples were assayed for the antigen-specific antibody at indicated times as described. ** indicates *P*<0.01 as compared with the VP2 control.

To further characterize the humoral adjuvanticity of Ag473 D1 domain, mice were immunized two times with VP2, D1-VP2 or VP2 + IFA, and the serum samples were collected for antibody detection. Compared to that in VP2 immune serum, a significantly (*P* < 0.01) higher level of the VP2-specific IgG antibody was detected in D1-VP2 immune serum at early as 7 dpi, but not in VP2 + IFA immune serum (Figure 3). From 14 dpi, the VP2-specific IgG antibody titers increased rapidly and reached to the highest levels by 28 dpi with a slight difference between the two adjuvant groups (Figure 3). These data suggest that fusion with Ag473 D1 domain can promote not only early antibody response, but stronger antibody response than incomplete Freund’s adjuvant (IFA) as well.

**Figure 4:**
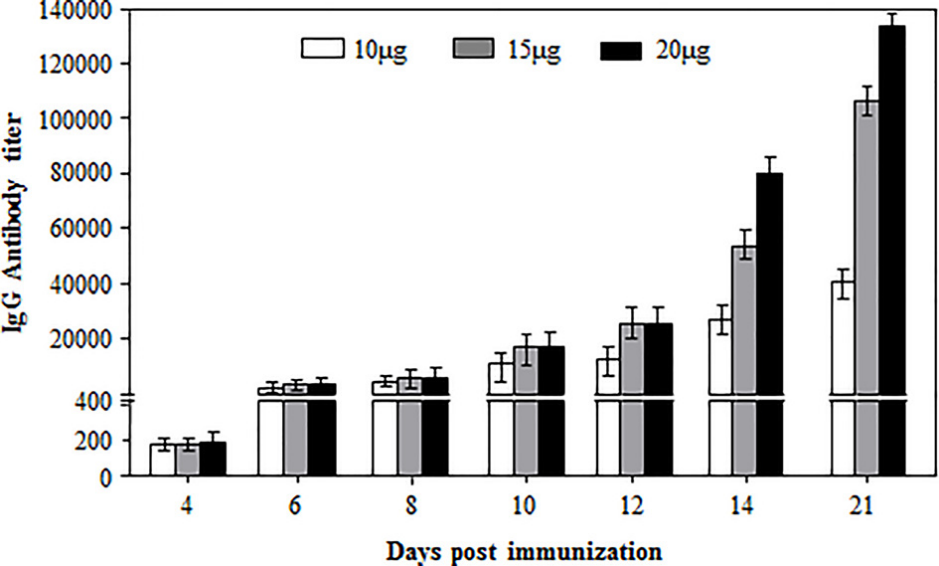
Dose-dependent antigen-specific total IgG antibody response in D1-VP2 immunized mice Mice were immunized two times with indicated doses of D1-VP2 fusion protein, and their serum samples were assayed for the antigen-specific antibody at indicated times as described.

To detect its dose-dependent antibody response, mice were immunized two times with 10, 15 or 20 µg of D1-VP2 fusion protein, and the serum samples were assayed for the VP2-specific IgG response. As early as 4 dpi, low titers of the antigen-specific IgG were detected in the three dose groups without significant difference (Figure 4). From 6 dpi, the antigen-specific IgG titers of the three dose groups increased rapidly in a dose-dependent manner and reached to the highest levels by 21 dpi (Figure 4). For a mouse immunization experiment, 50 µg of protein antigen with adjuvant is commonly used to induce a high level of antibody response. In the present study, however, as little as 10 µg of D1-VP2 fusion protein could induce a fairly high level of antibody response, confirming the stronger humoral adjuvanticity of Ag473 D1 domain.

**Figure 5:**
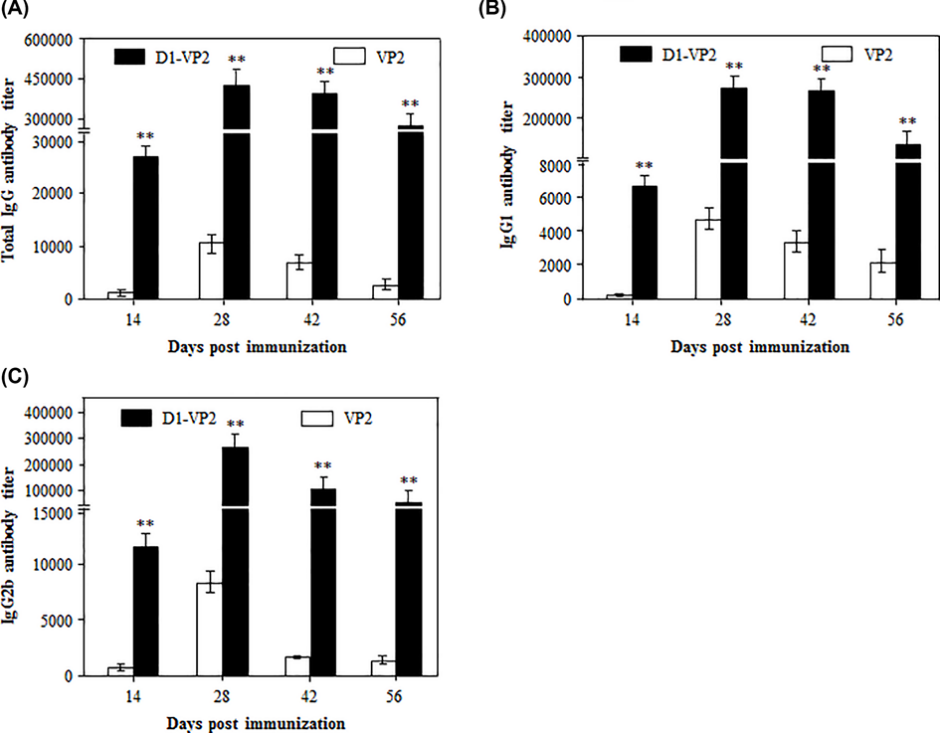
IgG isotypes stimulated by Ag473 D1 domain Mice were immunized two times with the indicated antigen formulations, and their serum samples were assayed for the antigen-specific total IgG (**A**), IgG1 (**B**) and IgG2b (**C**) at the indicated times as described. ** indicates *P*<0.01 as compared with the VP2 control.

To analyze the IgG isotypes stimulated by Ag473 D1domain, mice were immunized two times with 20 µg of VP2 or D1-VP2 fusion protein, and the serum samples were assayed for the antigen-specific total IgG, IgG1 and IgG2b. As shown in Figure 5A, the antigen-specific total IgG antibody in D1-VP2 immune serum reached to the highest titer by 28 dpi, followed by gradual decrease from 42 dpi, all of which were significantly higher than that in VP2 immune serum. Interestingly, a relatively high titer of IgG1, but not IgG2b, was detected in VP2 immune serum (Figure 5B,C). In contrast, similarly high levels of IgG1 and IgG2b antibodies were detected D1-VP2 immune serum (Figure 5B,C). These data suggest that Ag473 D1 domain could stumulate the balanced Th1(IgG2b)/Th2 (IgG1) antibody response.

## Conclusion

In the present study, we demonstrated that both cellular and humoral adjuvanticities of the three genetically fused TLR ligands were significantly stronger than that of co-administered TRL ligands. Among the three TLR ligands delivered in two different ways, fusion with Ag473 D1 domain exhibited the superior adjuvanticity and thus could be used as the universal strategy to convert non-lipoantigens into potent lipoimmunogens.

## Supplementary Material

Supplementary Figures S1-S3 and Tables S1-S2

## Abbreviations

ACK Lysis Buffer: erythrocyte lysate
Ag473: lipoprotein of *Neisseria meningitides*
APC: antigen-presenting cells
D1: D1 domain of Ag473
dpi: day post immunization
ELISA: enzyme-linked immunosorbent assay
FlaB: flagellin B
IFA: incomplete Freund’s adjuvant
MALDI-TOF/TOF: matrix-assisted laser desorption/ionization time-of-flight /time-of-flight
mHsp70: heat shock protein 70 of *Mycobacterium tuberculosis*
MS: mass spectrometry
TLR: toll-like receptor
VP2: capsid protein of IBDV

## References

[1] Moyle P.M. (2017) Biotechnology approaches to produce potent, self-adjuvanting antigen-adjuvant fusion protein subunit vaccines. Biotechnol Adv. 35, 375–389 10.1016/j.biotechadv.2017.03.005 28288861

[2] Ghimire T.R. (2015) The mechanisms of action of vaccines containing aluminum adjuvants: an in vitro vs in vivo paradigm. Springerplus 4, 181 10.1186/s40064-015-0972-0 25932368 PMC4406982

[3] Beutler B.A. (2009) TLRs and innate immunity. Blood 113, 1399–1407 10.1182/blood-2008-07-019307 18757776 PMC2644070

[4] St Paul M., Brisbin J.T., Abdul-Careem M.F. and Sharif S. (2013) Immunostimulatory properties of Toll-like receptor ligands in chickens. Vet. Immunol. Immunopathol. 152, 191–199 10.1016/j.vetimm.2012.10.013 23305711

[5] Chu C.L., Yu Y.L., Kung Y.C., Liao P.Y., Liu K.J., Tseng Y.T. et al. (2012) The immunomodulatory activity of meningococcal lipoprotein Ag473 depends on the conformation made up of the lipid and protein moieties. PLoS ONE 7, e40873 10.1371/journal.pone.0040873 22844415 PMC3402496

[6] Chen H.W., Liu S.J., Liu H.H., Kwok Y., Lin C.L., Lin L.H. et al. (2009) A novel technology for the production of a heterologous lipoprotein immunogen in high yield has implications for the field of vaccine design. Vaccine 27, 1400–1409 10.1016/j.vaccine.2008.12.043 19150476

[7] Leng C.H., Liu S.J., Chen H.W. and Chong P. (2015) Recombinant bacterial lipoproteins as vaccine candidates. Expert Rev. Vaccines 14, 1623–1632 10.1586/14760584.2015.1091732 26420467

[8] Lee S.E., Kim S.Y., Jeong B.C., Kim Y.R., Bae S.J., Ahn O.S. et al. (2006) A bacterial flagellin, Vibrio vulnificus FlaB, has a strong mucosal adjuvant activity to induce protective immunity. Infect Immun. 74, 694–702 10.1128/IAI.74.1.694-702.2006 16369026 PMC1346682

[9] Verma V., Tan W., Puth S., Cho K.O., Lee S.E. and Rhee J.H. (2016) Norovirus (NoV) specific protective immune responses induced by recombinant P dimer vaccine are enhanced by the mucosal adjuvant FlaB. J. Transl. Med. 14, 135 10.1186/s12967-016-0899-4 27184355 PMC4869196

[10] Ebrahimi S.M., Dabaghian M., Tebianian M. and Jazi M.H. (2012) In contrast to conventional inactivated influenza vaccines, 4xM2e.HSP70c fusion protein fully protected mice against lethal dose of H1, H3 and H9 influenza A isolates circulating in Iran. Virol. 430, 63–72 10.1016/j.virol.2012.04.015 22595444

[11] Xu Z. and Moyle P.M. (2018) Bioconjugation Approaches to Producing Subunit Vaccines Composed of Protein or Peptide Antigens and Covalently Attached Toll-Like Receptor Ligands. Bioconjug. Chem. 29, 572–586 10.1021/acs.bioconjchem.7b00478 28891637

[12] Pradhan S.N., Prince P.R., Madhumathi J., Roy P., Narayanan R.B. and Antony U. (2012) Protective immune responses of recombinant VP2 subunit antigen of infectious bursal disease virus in chickens. Vet. Immunol. Immunopathol. 148, 293–301 10.1016/j.vetimm.2012.06.019 22795186

[13] Grote A., Hiller K., Scheer M., Munch R., Nortemann B., Hempel D.C. et al. (2005) JCat: a novel tool to adapt codon usage of a target gene to its potential expression host. Nucleic Acids Res. 33, W526–W531 10.1093/nar/gki376 15980527 PMC1160137

[14] Li W., Wei Z., Qiao Z., Wu Z., Cheng L. and Wang Y. (2013) Proteomics analysis of alfalfa response to heat stress. PLoS ONE 8, e82725 10.1371/journal.pone.0082725 24324825 PMC3855785

[15] Duthie M.S., Windish H.P., Fox C.B. and Reed S.G. (2011) Use of defined TLR ligands as adjuvants within human vaccines. Immunol. Rev. 239, 178–196 10.1111/j.1600-065X.2010.00978.x 21198672 PMC5872835

[16] Gnjatic S., Sawhney N.B. and Bhardwaj N. (2010) Toll-Like Receptor Agonists Are They Good Adjuvants? Cancer J. 16, 382–391 10.1097/PPO.0b013e3181eaca65 20693851 PMC2922045

[17] Mosmann T.R., Cherwinski H., Bond M.W., Giedlin M.A. and Coffman R.L. (1986) Two types of murine helper T cell clone. I. Definition according to profiles of lymphokine activities and secreted proteins. J. Immunol. 175, 5–14 15972624

[18] Chong P., Huang J.H., Leng C.H., Liu S.J. and Chen H.W. (2015) Recombinant Lipoproteins as Novel Vaccines with Intrinsic Adjuvant. Adv. Protein Chem. Str. Biol. 99, 55–74 10.1016/bs.apcsb.2015.03.003 26067816

[19] Hajam I.A., Dar P.A., Shahnawaz I., Jaume J.C. and Lee J.H. (2017) Bacterial flagellin-a potent immunomodulatory agent. Exp. Mol. Med. 49, e373 10.1038/emm.2017.172 28860663 PMC5628280

[20] Gupta S.K., Deb R., Dey S. and Chellappa M.M. (2014) Toll-like receptor-based adjuvants: enhancing the immune response to vaccines against infectious diseases of chicken. Expert Rev. Vaccines 13, 909–925 10.1586/14760584.2014.920236 24855906

