# Supplementary material for: Superior adjuvanticity of the genetically fused D1 domain of Neisseria meningitides Ag473 lipoprotein among three Toll-like receptor ligands
Source: Biosci Rep. 2020 Apr 7;40(4):BSR20193675. doi: 10.1042/BSR20193675 (PMC7138904; doi:10.1042/BSR20193675)
Supplement: Supplementary Figures S1-S3 and Tables S1-S2 [file BSR-2019-3675_supp.pdf]

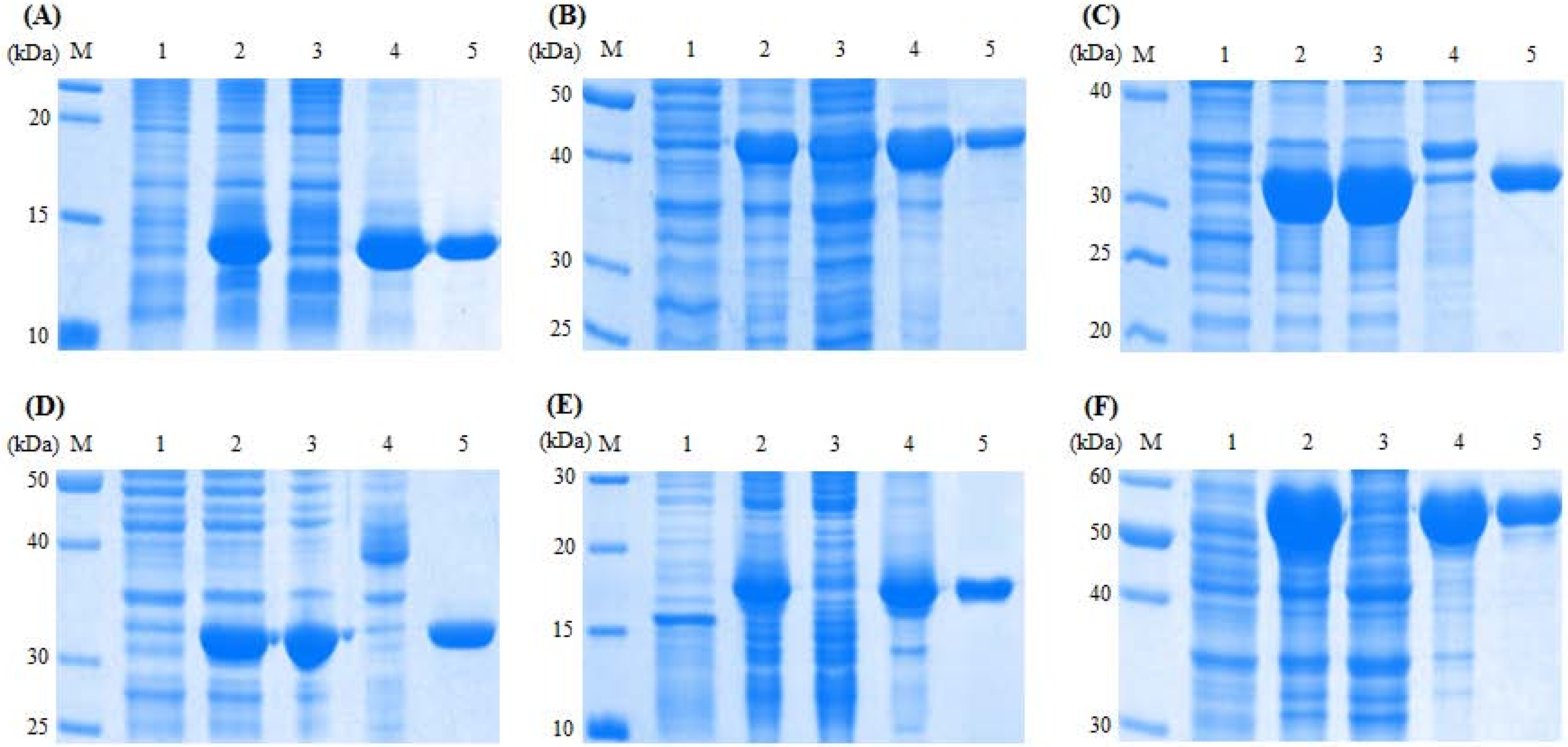

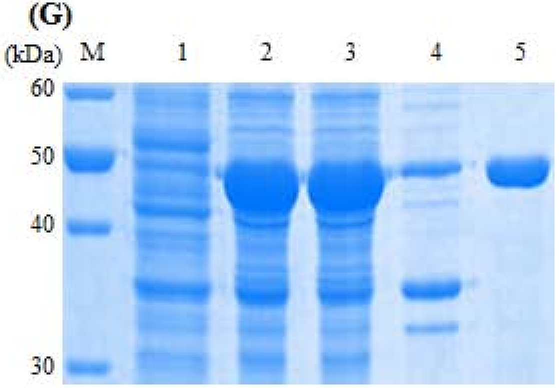

Supplementary Figure 1. SDS-PAGE analysis of recombinant protein expression and purification. The expression of recombinant proteins VP2 (**A**), FlaB (**B**), mHsp70 (**C**), Ag473 (**D**), D1-VP2 (**E**), FlaB-VP2 (**F**) and VP2-mHsp70 (**G**) in *E. coli* strain BL21 (DE3) were induced with 0.2 mM IPTG for 6 h at 37 ℃. The soluble and insoluble recombinant proteins were purified under native and denatured conditions, respectively, by nickel affinity chromatography. The cell lysates before (1) and after (2) IPTG induction, the soluble (3) and insoluble (4) fractions of centrifuged cell lysates and purified proteins (5) were analyzed by 12% SDS-PAGE.

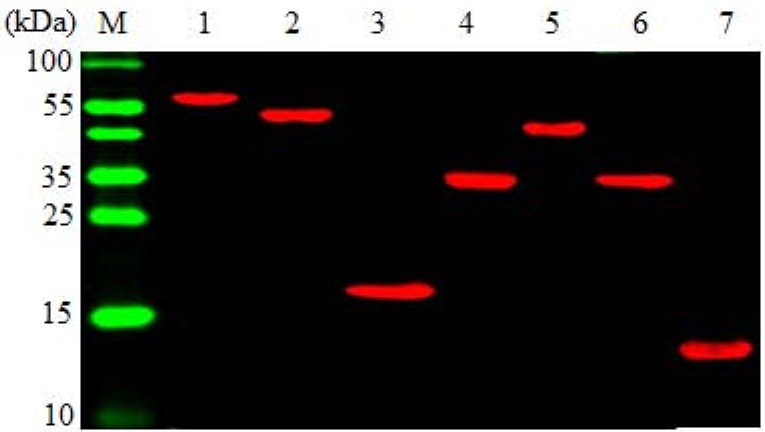

Supplementary Figure 2. Western blotting analysis of purified proteins. The purified proteins were separated by 12% SDS-PAGE. After transferred to nitrocellulose membrane, the proteins were identified using anti-His mAb as the first antibody and DyLight 800-labeled goat anti-mouse IgG as the second antibody. 1-7 indicate FlaB-VP2, VP2-mHsp70, D1-VP2, Ag473, FlaB, Hap70, and VP2, respectively.

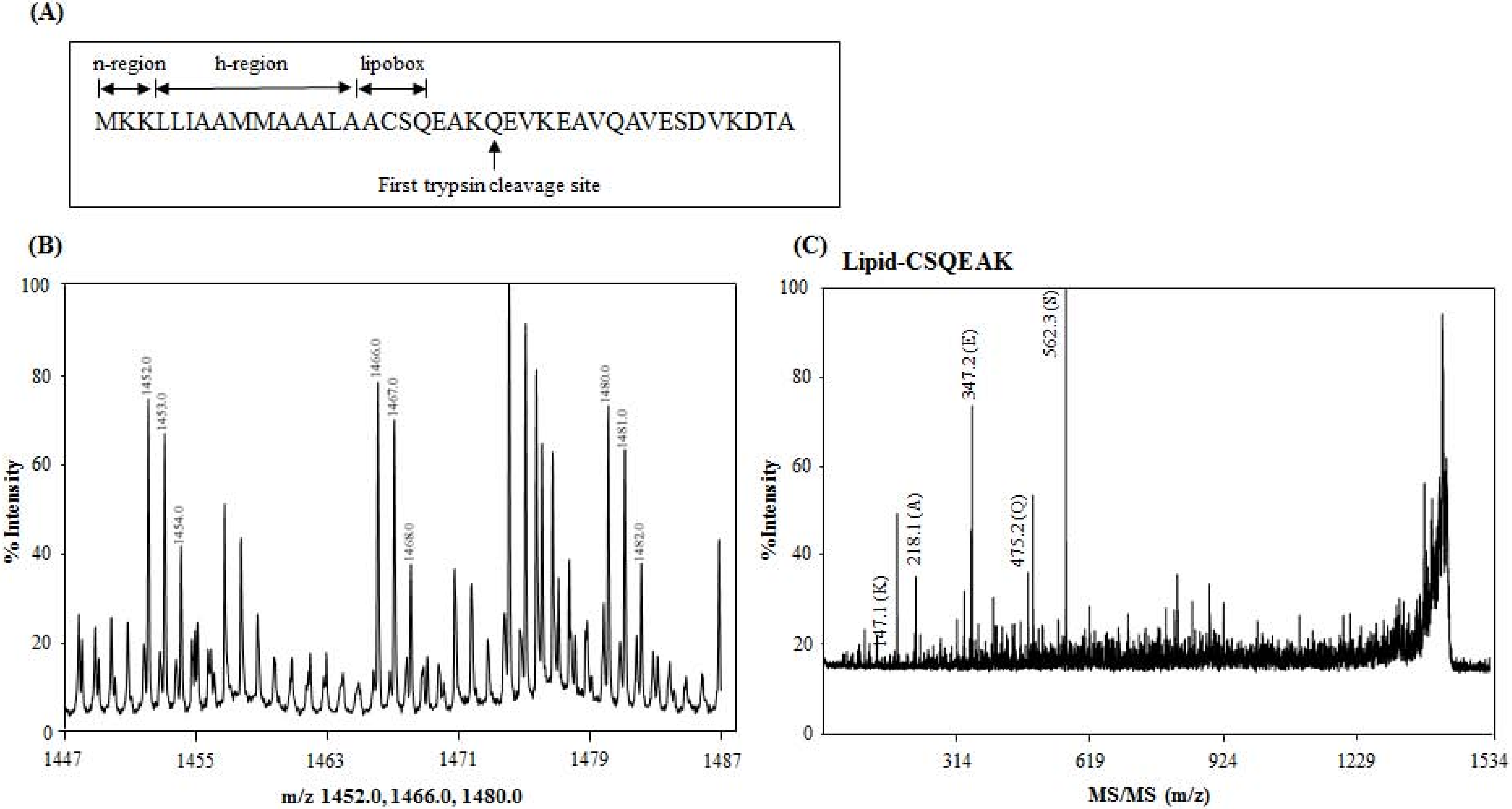

Supplementary Figure 3. MALDI-TOF/TOF analysis of lipid modification of D1-VP2 fusion protein. (**A**) The structure and amino acid sequence of Ag473 D1 domain used for VP2 fusion protein expression. (**B** and **C**) The N-terminal tryptic segment and the corresponding lipid-modified sequence of D1-VP2 fusion was analyzed by AB5800 MALDI-TOF/TOF mass spectrometry.

Suplementary Table 1 The PCR primer pairs used in this study

Gene	Primer pair	Sequence (5'→3')	Amplicon (bp)	GenBank
VP2	Sense	AT*CATATG*CTGCTGATGCCGACCACCG	366	FJ848772
	Antisense	AT*CTCGAG*AACGTCGGTCAGTTCAGAC		
Ag473 D1	Sense	GC*CATATG*AAAAAACTGCTGATCGCTGC	117	WP_002220617.1
	Antisense	AT*GGATCC*AGCGGTGTCTTTAACGTCAG		
FlaB	Sense	GC*CATATG*GCTGTTAACGTTAACACCA	1128	WP_011078329.1
	Antisense	GG*CTCGAG*ACCCAGCAGAGACAGAGCA		
Hsp70	Sense	GC*CATATG*GAAGTTAAAGACGTTCTGC	801bp	ACE79189.1
	Antisense	AT*CTCGAG*TTTAGCTTCACGACCGTCG		

The restriction sites for gene cloning are underlined.

Suplementary Table 2 The tryptic segments of D1-VP2 fusion protein analyzed by PeptideMass

Position	Mass	Peptide sequence
1-6	665.2923	CSQEAK
7-10	503.2824	QEVK
11-21	1174.5950	EAVQAVESDVK
22-44	2330.1431	DTAGSLLMPTTGPASIPDDTLEK
45-48	526.3096	HTLR
49-90	4396.1407	SETSTYNLTVGDTGSGLIVFFPGFPGSIVGAHYTLQSNGNYK
91-109	2248.0525	FDQMLLTAQNLPASYNYCR
114-118	575.3511	SLTVR
119-149	3139.6157	SSTLPGGVYALNGTINAVTFQGSLSELTDVK
150-157	847.5400	ALKPPPPK
159-166	1083.4979	LEHHHHHH

The amino acid sequence of D1-VP2 fusion protein was analyzed using peptide characterization software PeptideMass (https://web.expasy.org/peptide_mass).
